# Recent Trends in Exhaled Breath Diagnosis Using an Artificial Olfactory System

**DOI:** 10.3390/bios11090337

**Published:** 2021-09-14

**Authors:** Chuntae Kim, Iruthayapandi Selestin Raja, Jong-Min Lee, Jong Ho Lee, Moon Sung Kang, Seok Hyun Lee, Jin-Woo Oh, Dong-Wook Han

**Affiliations:** 1 BIO-IT Foundry Technology Institute, Pusan National University, Busan 46241, Korea; chuntae1122@gmail.com (C.K.); rajaselestin@gmail.com (I.S.R.); 2 School of Nano Convergence Technology, Hallym University, Chuncheon 24252, Korea; jongminlee1984@gmail.com; 3 Daan Korea Corporation, Seoul 06252, Korea; nunssob@gmail.com; 4 Department of Cogno-Mechatronics Engineering, Pusan National University, Busan 46241, Korea; mskang7909@gmail.com (M.S.K.); seokhyun2285@gmail.com (S.H.L.); 5 Department of Nanoenergy Engineering, Pusan National University, Busan 46241, Korea

**Keywords:** artificial olfactory system, health monitoring, exhaled breath diagnosis, volatile organic compounds, gas sensor, electronic nose

## Abstract

Artificial olfactory systems are needed in various fields that require real-time monitoring, such as healthcare. This review introduces cases of detection of specific volatile organic compounds (VOCs) in a patient’s exhaled breath and discusses trends in disease diagnosis technology development using artificial olfactory technology that analyzes exhaled human breath. We briefly introduce algorithms that classify patterns of odors (VOC profiles) and describe artificial olfactory systems based on nanosensors. On the basis of recently published research results, we describe the development trend of artificial olfactory systems based on the pattern-recognition gas sensor array technology and the prospects of application of this technology to disease diagnostic devices. Medical technologies that enable early monitoring of health conditions and early diagnosis of diseases are crucial in modern healthcare. By regularly monitoring health status, diseases can be prevented or treated at an early stage, thus increasing the human survival rate and reducing the overall treatment costs. This review introduces several promising technical fields with the aim of developing technologies that can monitor health conditions and diagnose diseases early by analyzing exhaled human breath in real time.

## 1. Introduction

**Figure 1:**
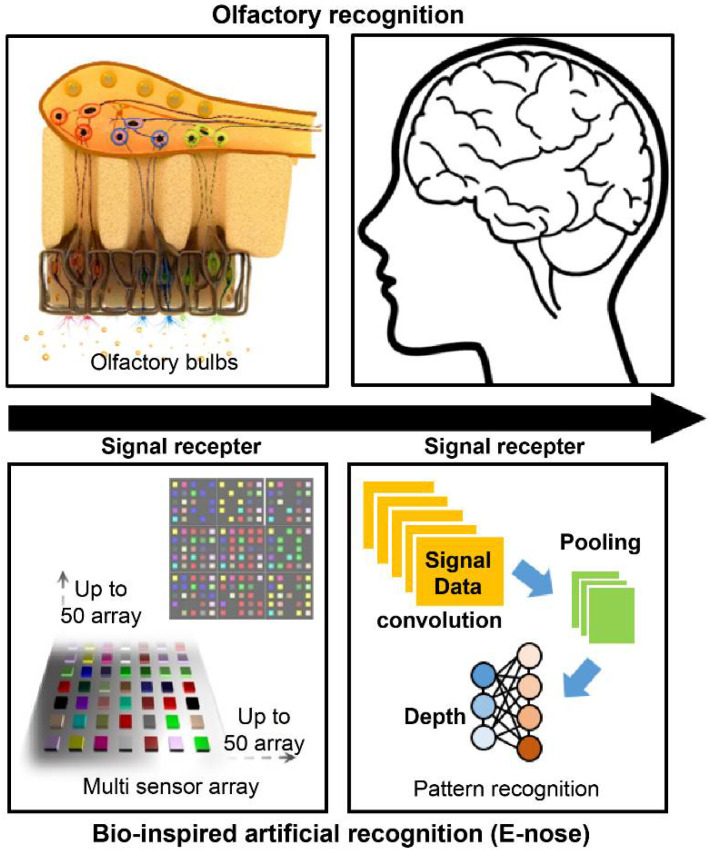
Concept of an artificial nose (e-nose) system based on the olfactory perception pathway.

The olfactory sense is the oldest human sense. Therefore, humans tend to analyze the environment by sniffing [1]. Various studies have been conducted to mimic the sensory cognitive mechanism, and research on sensor systems focusing on olfactory cognitive models has attracted significant attention [2]. Sniffing is a complicated process as there are over 400 species of olfactory receptor gene, and signals through various receptors are comprehensively determined in the brain to provide information about smell and human cognition [3]. The concept of an “artificial nose” is based on a technology that grasps information about odors and uses them as data. In other words, it is an electronic system technology that analyzes the state and composition of a substance through smell. Figure 1 shows the concept of an artificial nose system inspired by the olfactory perception pathway. A biomimetic gas detection platform can be designed by constructing a nanosensor array based on the olfactory receptor tissue and by combining a data processing technology through pattern analysis of signal data.

Persaud et al. proposed the concept of using different types of sensors as arrays and applying unique signal patterns to specific odors to enhance the selectivity, reliability, and accuracy of gas detection systems [4]. The developed system was called an electronic nose (e-nose). All corresponding technologies associated to this system are called e-noses. The principle of the e-nose is similar to that of the human olfactory mechanism. That is, the odor recognition is based on the reaction of the smell factor and olfactory receptors. The e-nose reacts with odor substances using sensor unit devices instead of olfactory receptor cells as receptors, and analyzes patterns using a computer instead of the brain [5].

In the 21st century, the e-nose technology has evolved significantly along with the development of NT/IT technologies. Low-cost, high-performance sensor devices have been developed on the basis of various nanobiosensor technologies, and the data processing analysis technologies have been improved owing to the advent of artificial intelligence (AI)-integrated technologies. The evolution of AI and big data processing technologies has subsequently led to the development of high-level e-nose technologies that utilize a large number of sensor arrays [6]. The current artificial-nose technology can sniff a smell that cannot be smelled by humans using a sensor unit with a sensitivity of parts-per-billion level [7]. Recently, this technology has been used in various fields such as chemistry, medical care, food quality management, and military industry [8,9,10,11]. In the case of conventional gas sensors, the information on the gas concentration level is acquired by quantitatively analyzing specific chemicals. The e-nose sensors do not require such an extensive process and have been increasingly used because of their simple structure that recognizes patterns for only specific odors through a database.

This paper introduces several research cases that analyze the smell of breath exhaled by humans using an artificial olfactory system; in many cases, an e-nose is used. When a person is diagnosed with a certain disease, various volatile organic compounds (VOCs) are produced in vivo owing to metabolic disorders or free radicals [12,13,14]. Such VOCs serve as unique biometric data depending on the specificity, stage, and condition of the disease. In other words, as the conditions under which VOCs occur depend on the particular disease and its various stages, they are classified as “fingerprints” of the disease conditions. Therefore, the analysis of the exhaled gas can provide information on the physiological and health status of an individual. This information can be used for the early diagnosis of many diseases during the induction stage [15]. In this study, we describe the overall concept of disease diagnosis using human respiratory gas, which has gained significant attention in recent years. Furthermore, we describe the trend of human respiratory gas analysis technologies used for diagnosing such diseases and analyze future scenarios of human respiratory gas disease analysis.

## 2. Exhaled Breath Diagnosis

In conventional approaches, diseases were distinguished on the basis of the breath of patients. Recent reports indicate that dogs that are trained to detect various drugs and explosives by smell can also distinguish cancer patients from healthy people. The olfactory analysis method is a natural approach that is characterized by many possibilities. The current drunk-driving control technologies are based on the blood alcohol concentration measurement, and human breath gas analysis methods are being actively researched. A majority of this research is directed toward the analysis of respiratory diseases such as lung cancer and asthma [16,17]. Once the relationship between a disease and breath gas components is identified, the disease diagnosis based on the exhaled breath analysis is expected to emerge as a future disease diagnostic method [18].

In general, the diagnostic methods using expensive analytical equipment by collecting samples of patients’ blood, tissues, etc. require a time-consuming sample collection process and a skilled operator owing to complex protocols. In addition, such methods can only be performed in a limited number of places such as hospitals. In contrast, disease diagnosis using the analysis of breathing gas can be easily performed by operators and users, and the identification process of the analysis results can be verified in real time. Disease diagnosis by analyzing breath gas is considered to be an innovative non-invasive diagnostic method. Breath gas diagnosis has potential advantages over other diagnostic methods such as blood sampling, urine sampling, biopsy, endoscopy, and imaging. First, it is a completely non-invasive approach that allows the development of a user-friendly, simple, and intuitive diagnostic platform [19]. Second, the sample collection in this method is advantageous because it has superior processing when compared to serum or urine sampling [20]. Finally, it is the most convenient method as it does not pose the problem of bio-hazardous specimens within the current regulations.

Breath gas diagnosis is based on the physiological phenomenon of gas exchange occurring in the alveoli. Human blood contains chemicals that reflect physiological phenomena and metabolic conditions in the human body. Low-molecular organic compounds are contained in the human exhaled breath through the lungs during the respiratory process and are released out of the body [21]. The health conditions of the human body are reflected by various VOCs contained in breathing gases that undergo various biological reactions [22]. Blake et al. [23] reported that various metabolic diseases and pathological conditions could be observed in relation to the concentration increase of the aforementioned VOCs in the exhaled breath gas. These are associated with the respiratory diseases, such as cardiovascular disease (CVD), diabetes, asthma, bacterial infertility and inflammatory disease, cancer, COPD, and Alzheimer’s disease, as well as with other diseases, according to the results of a recent breath analysis. In addition, a number of studies have been conducted on the applicability of diagnostic technologies using these VOC gases.

Table 1 summarizes the biomarker VOCs for each disease that can be used for its diagnosis and the currently used corresponding diagnostic methods. Although it cannot be used as a replacement for the conventional diagnostic method, it has the potential to serve as a portable diagnostic device capable of periodic self-diagnosis, which solves the problems of location and equipment. Through an accurate and efficient analysis of specific biomarker VOCs present in the breath, we demonstrate the possibility of developing a new selective self-diagnosis platform.

### 2.1. Diabetes

Diabetes is an abnormal carbohydrate metabolic disease that is characterized by hyperglycemic symptoms [39]. It is classified as either type 2 or type 1 depending on the problems with insulin secretion or various degrees of peripheral nerve resistance to insulin action [40,41]. Currently, the most widely applied diabetes diagnostic methods include the fasting plasma glucose, oral glucose tolerance, and glycated hemoglobin [42]. Diabetes is a condition of abnormal blood sugar in patients, and the complications that follow place a considerable burden on clinical and public health. Accordingly, an effective intervention that detects the glucose abnormalities early and prevents progression from prediabetes to diabetes is of utmost importance. Diabetes should be thoroughly managed by the patient through a self-diagnosis. The primary self-diagnosis method is based on glucose level measurement using existing blood collection methods [43]. Although the low-cost diagnostic technology is currently in use, users’ reluctance to collect blood remains to be addressed. If low-cost and efficient non-invasive diagnostic methods are developed in the near future, it is expected to be an innovation in the health care market related to diabetes.

Galassetti et al. [23] presented a correlation between ethanol and acetone in exhaled breath, which is related to serum glucose levels. In the case of patients suffering from diabetic ketoacidosis, a study showed that the acetone concentration in the exhaled gas increases to hundreds of ppmv [44]. When the blood sugar levels remain high over long periods of time, the fatty and amino acids are burned to produce energy. The ketone body produced in the body is a 3-carbon ketone body derived from the oxidation of non-esterified fatty acids, and is found in the state of hydroxyacetone (1-hydroxyacetone) and 1,2-propanediol (PPD) through acetoacetate decarboxylase [45]. The ketone bodies are stored in the blood and thus, lower the pH level. Therefore, glucose cannot be used as an energy source for untreated diabetic patients. As a result, the ketone bodies are produced as by-products and energy sources when fat is broken down instead of glucose. High levels of ketones are produced as a result of low insulin levels in diabetic ketoacidosis [46]. Therefore, the exhaled acetone can be detected in patients with diabetic ketoacidosis and a high-fat diet [25]. These research results can be used for developing a diabetes diagnosis technology for acetone level monitoring in exhaled gas. The introduction of a simple and low-cost measurement technology could enable its use in a wide range of selective testing methods and reduce patient discomfort or pain during blood collection.

### 2.2. Various VOCs Derived from Inflammatory Diseases

In addition to diabetes, a few other diseases are expected to be diagnosed through the analysis of VOCs in exhaled breath gas. In vivo immune responses that are caused by bacterial infections or inflammatory reactions produce various types of VOC in the body. The pathological problems can interfere with normal metabolism, and abnormal chemical reactions can cause the detection of some VOCs in the exhaled gas [44]. By detecting volatile chemicals such as ammonia, nitrogen oxides, and hydrogen sulfide, the cause of pathological reactions can be analyzed and implemented in the diagnostic technology [47]. Mathew et al. [46] reviewed various types of VOC caused by metabolic processes in the body and metabolic disorders caused by various pathological reactions, and suggested the possibility of the implementation of a breath diagnosis technology. The level of ammonia (NH3) in breath gas can be used as an important biomarker. In the human body, ammonia is produced during protein metabolism and is regulated through the urea circuit owing to its toxicity [48]. In the case of liver and kidney problems, ammonia levels increase [49]. In addition, bacteria such as H. pylori, which cause peptic ulcers from the oral cavity to the duodenum, excrete ammonia gas and hydrogen sulfide through metabolic processes [50]. Nitrogen monoxide and nitrogen oxide are important signaling substances produced in the human body. Within the respiratory system, NO regulates the tension of blood vessels and bronchi (promotes the expansion of blood vessels and airways), promotes the coordinated beating of ciliated epithelial cells, and acts as an important neurotransmitter of non-adrenergic, non-cholinergic neurons running in the bronchi. Diseases related to inflammation can be mainly analyzed through the concentration of NO in the breath [51,52,53,54,55,56,57,58]. Currently, a technology that can measure NO in the respiratory tract using laser analysis sensors is being developed and used as a reference for diagnosing inflammatory diseases [59].

Isoprene is a unit molecule that forms cholesterol and is involved in cholesterol metabolism. Its concentration can be used as a sensitive and non-invasive metric for analyzing various metabolic effects in the human body [60]. Among the cholesterol metabolic diseases, CVDs and hypertension are categorized as representative diseases with a very high risk, and the patient needs constant management through periodic and voluntary diagnosis [61,62]. The currently used self-diagnosis method is the patient family history analysis and periodic monitoring of the blood pressure level [63,64]. Research on the classification of exhaled breath components of patients based on various levels of VOC other than hydrocarbons in the breath is being actively conducted [35]. The following information is from a review by Mathew et. al. [46] for journal diagnostics in 2015. In the case of hydrocarbons such as ethane and pentane, it is caused by oxidation of lipid components in cells [65]. This component is found when a problem occurs in the metabolism of lipid components, and is advantageously released through breath gas owing to its low solubility. It is mainly associated with respiratory obstructive diseases caused by inflammation, such as asthma, COPD, obstructive sleep apnea, and ARDS [66,67,68,69,70]. The hydrocarbon molecules are biomarkers of oxidative stress, and pentane and ethane concentrations increase owing to physical and mental stress [71,72,73]. It shows a significant difference in concentration in sepsis or SIRS patients [70] and can be used in the diagnosis of inflammatory bowel disease, sleep apnea, cancer, and ischemic heart disease [74,75,76,77].

Asthma [78] and COPD [79], which are obstructive respiratory diseases, should be detected early and prevented from worsening through rapid response. According to the currently widely known medical manual, patients with active asthma need periodic checkups every one to six months, depending on the severity of asthma. Asthma symptoms are diagnosed through equipment that detects lung function, such as spirometry, in hospitals [80]. In addition, lung capacity indicators are periodically managed at home using a personal peak flow meter (which approximately costs $20) [81]. On the basis of pathological research results on the levels of various VOC biomarker substances, such as NO, in the patient’s exhaled strain and hydrocarbons, research on inflammatory asthma patients breathing diagnosis using breath gas analysis is actively underway [82,83,84].

### 2.3. Cancer

Conventionally, cancer diagnostics include genetic, epigenetic, proteomic, and glycomic biomarker screening, as well as some non-invasively collected biofluids [85,86,87,88,89]. In the case of cancer, early detection may increase the chance of complete recovery [90]. If a self-diagnosis technology enabling easy breathing gas analysis is developed, health and medical expenses for cancer treatment can be reduced, and average life expectancy can be increased. A malignant tumor, commonly known as cancer, is a disease wherein cell mutations are caused by several risk factors to identify the cause, which leads to abnormal cell growth and metastasis. It is reported that approximately 100 types of cancer affect the human body.

Because the reactions of cancer cells in the body are complex, various types of VOCs can be used as exhaled breath biomarkers. The first study on cancer diagnosis was conducted for lung cancer, which is a malignant tumor of the lung tissue through direct gas exchange. O’Neil et al. [91] conducted a study to select candidate groups of biomarker VOCs through GC/MS by collecting breath samples from eight lung cancer patients. It was found that among the 386 component gases that were detected with this technology, 45 components were at >75% occurrence level and 28 components were at >90% occurrence level. The research results were significant because they could distinguish between normal samples and patient respiration gas using a classification process via a computer program. Since then, many research results related to the analysis of biomarker VOCs that are common in lung cancer patients have been published [92,93].

Phillips et al. published a study on biomarker VOCs produced by the intracellular oxidative stress caused by breast cancer [94]. Oxidative stress is the process of oxidizing biologically important molecular substances, including DNA and proteins, when an increased amount of reactive oxygen species (ROS) is leaked into the cytoplasm in mitochondria [95]. This causes the decomposition of fatty acids and the peroxidation of lipids by abundant oxygen radicals [96,97]. Breast cancer patients and control patients were classified using SPSS treatment, and higher negative value (NPV) and lower positive value (PPP) were derived, respectively, as compared with the results of the screening mammograms. Kumar et al. published a selected ion flow tube mass spectrometry (SIFT-MS) analysis of exhaled breath for VOC profiling of esophagogastric cancer [98]. In approximately 17 VOC species, the concentrations of hexanoic acid, phenol, methyl phenol, and ethyl phenol differed statistically from those in the positive control group.

Until recently, studies on exhaled breath gas analysis for various cancer disease models and classified patient and control groups have been published steadily. If the integrated analysis sensor array technology that can classify breathing gas samples according to the composition of VOCs is used, a new concept diagnosis technology can be developed for various disease models described above. Furthermore, if breath diagnosis technology is deployed as a low-cost artificial-nose platform consisting of simple chemical sensor units, it will be possible to design self-diagnostic devices that can be used by individuals in real time at home. This technology could be an excellent innovation in health and medical industry.

## 3. Nanosensor Array E-Nose for Exhaled Breath Diagnosis

The sensor array technology has recently been widely applied in disease diagnosis for exhaled breath gas analysis because it is efficient for analyzing multiple VOCs, including human breath gases [99]. Because exhaled breath gas samples have different compositions of the compounds, their individual analysis using conventional devices, such as existing analytical instrument GC-MS, is limited. The sample component profile can be patterned and recognized using the nanosensor array technology. The artificial nose, which can analyze the components of real-time breathing based on a simple system, is a novel technology that can be used for disease self-diagnosis in healthcare. In terms of the practical application and commercialization of e-nose sensors, low cost, ease of use, and miniaturization are key factors. To meet these requirements, sensor technologies are being developed based on the mechanisms derived from the unique characteristics of various materials [100]. In this section, we discuss electrochemical sensors (metal oxide (MO) nanomaterial-based e-nose sensors) and colorimetric sensors (metal-containing dye sensor arrays and functional phage sensor arrays).

Table 2 summarizes representative nanosensor technologies that can be used for diagnosis based on recent gas detection methods. We aim to introduce an electrochemical sensor method and a technology to detect a small amount of gas mixture using a color sensor. To detect various VOCs present in exhaled breath, artificial olfactory models have been developed using a variety of nanosensor technologies that distinguish specific gas molecules. These technologies are expected to be applicable to disease diagnosis in the future.

### 3.1. Metal Oxide-Based Electrochemical Sensor Array for Disease Diagnosis

Metal oxide (MO) nanoparticles are promising candidates for sensor element design owing to their remarkable physicochemical properties, adjustable surface properties, and good stability [110]. These nanoparticles have a high density of trapped charged oxygen species (O^2−^, O^−^, and O^2−^), creating a surface charged layer in the sensor element. When the reacting gaseous molecules adsorb oxygen ions on an MO surface, they alter the surface-trapped charge density [111,112]. The number of oxidation states used for gas sensing at the parts-per-billion level can be controlled by the nanoparticle size, shape, and composition. Many transition metal elements such as Fe, Co, Ni, Mn, Al, and Cu have been used as dopants for improving the electrical and optical characteristics of MOs and for enhancing their sensitivity to gases [113].

**Figure 2:**
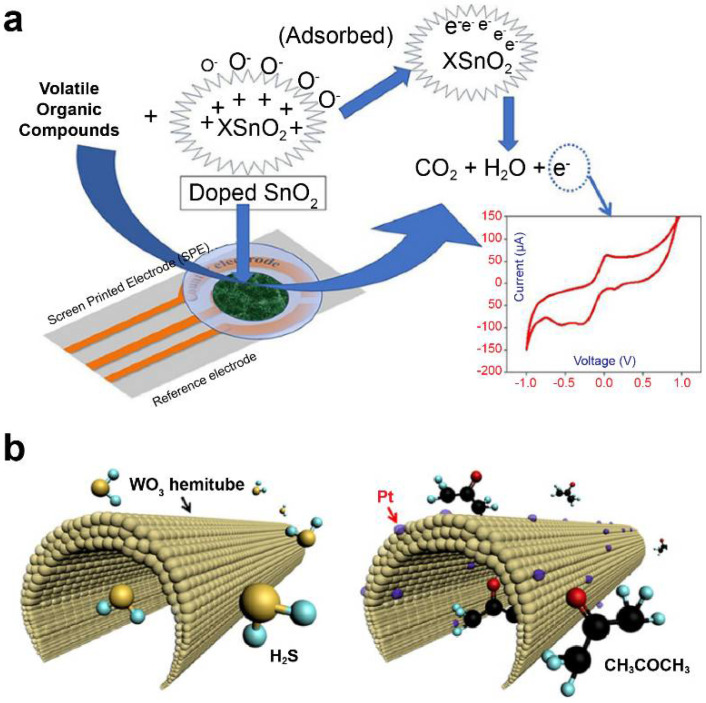
Metal–oxide nanomaterial-based electrochemical sensors. (**a**) Doped SnO_2_ nanomaterial sensor for lung cancer diagnosis. Data reproduced from Ref. [101]. Copyrights ACS 2020. (**b**) Diabetes diagnosis model using polycrystalline WO_3_ hemi-tube for H_2_S, selective acetone detecting sensors. Data reproduced from Ref. [116]. Copyrights ACS 2013.

Khatoon et al. [101] doped Co and Ni with tin oxide (SnO_2_) using a sol–gel method and investigated it as a sensor material for e-nose development. (Figure 2a) They applied an MO-based screen-printed electrode as the working electrode to determine the levels of 1-propanol and isopropyl alcohol in cyclic voltammetry. Furthermore, Ni-SnO_2_ and Co-SnO_2_ were found selective to 1-propanol and isopropyl alcohol, respectively, among other investigated VOCs (acetone, toluene, formaldehyde, 2-butanol, and ethyl acetate). Liu et al. [114,115] developed various CeO_2_-based gas sensors attached with different MMnO_3_ (M: Sr, Ca, La, and Sm) sensing electrodes and conducted a comparative study for detecting acetone gas. CeO_2_-MMnO_3_ compounds were prepared using a simple sol–gel method.

Nanostructured materials with various morphologies, including nanorods, nanowires, nanosheets, and nanofibers, have been developed for e-nose applications because their large surface area-to-volume greatly facilitates the conversion of gas response into electric signals. Srinivasan et al. [104] developed an acetone sensor using nanostructured Co_3_O_4_ thin films for the detection of diabetic ketoacidosis (DKA). The presence of acetone at a trace level (1.8 ppm) in human exhaled breath signifies the presence of DKA from a diagnostic perspective.

The acetone level of the exhaled breath air of type-2 diabetes mellitus patients exceeded 1.71 ppm, whereas that of type-1 diabetes patients was 2.19 ppm. It is known that the concentration of acetone is directly proportional to metabolic disorders in humans. The Occupational Safety and Health Administration states that the allowable human exposure level of acetone is 1000 ppm in industries. Different nanostructured cobalt oxide sensing elements were synthesized using a spray pyrolysis method at different deposition temperatures (473 to 773 K) [117]. It was found that the nanostructured material was more sensitive to acetone when analyzing different solvents such as acetone, EtOH, NH_3_, xylene, toluene, and acetaldehyde. The sensor fabricated at 773 K exhibited a response of 235 toward acetone (50 ppm) at room temperature.

Furthermore, the sensor showed a limit of detection of 1 ppm, which is lower than the minimum threshold level of DKA. Ren et al. synthesized four different Fe-doped TiO_2_ thin films on Ti plates using the microarc oxidation technique to measure the level of ethanol gas [118]. The Fe-doped TiO_2_ thin films fabricated by introducing 0.5 mM K_4_(FeCN)_6_·3H_2_O into 0.5 M Na_3_PO_4_ showed a better sensitivity to ethanol gas with a response of 7.9. This value was significantly larger than the responses of other samples (less than 5.2, at 275 °C) prepared with different formulations of K_4_(FeCN)_6_·3H_2_O and Na_3_PO_4_. Li et al. synthesized pristine SnO_2_ and Er-SnO_2_ nanobelts using the thermal evaporation method. When analyzing 100 ppm of various gases including formaldehyde, ethanediol, ethanol, and acetone at temperature ranging from 150 °C to 260 °C, it was found that the Er-SnO_2_ nanobelt was more sensitive to formaldehyde gas than other gases. The experimental results revealed that the gas response of a single Er-SnO_2_ nanobelt device was 9, with response and recovery times of 17 s and 25 s, respectively [119].

Wang et al. [120] also prepared SnO_2_ and SnO_2_/NiO electrospun nanofibers, which were subsequently subjected to thermocompression and calcination processes. A fabricated SnO_2_/NiO sensor was more sensitive to ethanol vapor than to other gases such as H_2_S, CO, NH_3_, and acetone. A SnO_2_/NiO nanofiber exhibited a higher Ra/Rg value (27.5) than a pristine SnO_2_ nanofiber (2.4) on sensing 100 ppm of ethanol and showed average response and recovery times of 2.9 s and 4.7 s, respectively. Li et al. [121] synthesized porous Nb_2_O_5_-TiO_2_ n–n junction nanofibers with different Nb molar ratios by electrospinning.

Choi et al. used detection sensors to demonstrate promising clinical applications for diagnostic purposes through correlation analysis between exhalation components and specific diseases [116]. They utilized 1D fibers with uniformly applied platinum nanoparticle catalysts on a porous tin oxide (SnO_2_) sensor material surrounded by layers of thin shells (Figure 2b). When acetone gas was adsorbed on the surface of the material under study, it was applied to a sensor for detecting acetone concentration at approximately 120 ppb, which resulted in a change in the electrical resistance value. The developed nanofiber sensor increased the resistance of the material by up to six times at a concentration of acetone of 1000 ppb, allowing the diagnosis of diabetes. According to another study, wherein trained dogs diagnosed lung cancer, on average, toluene was detected approximately at an 80% accuracy [122]. This sensor was found to detect toluene with an accuracy of approximately 70%. Active research is being conducted on using multisensor arrays for various-disease diagnosis, such as lung cancer and diabetes. An increased number of research studies related to the detection of VOCs based on electrochemical sensors via the change in the resistance of MOs are being published.

### 3.2. Colorimetric Sensor Array for an Artificial Nose System

**Figure 3:**
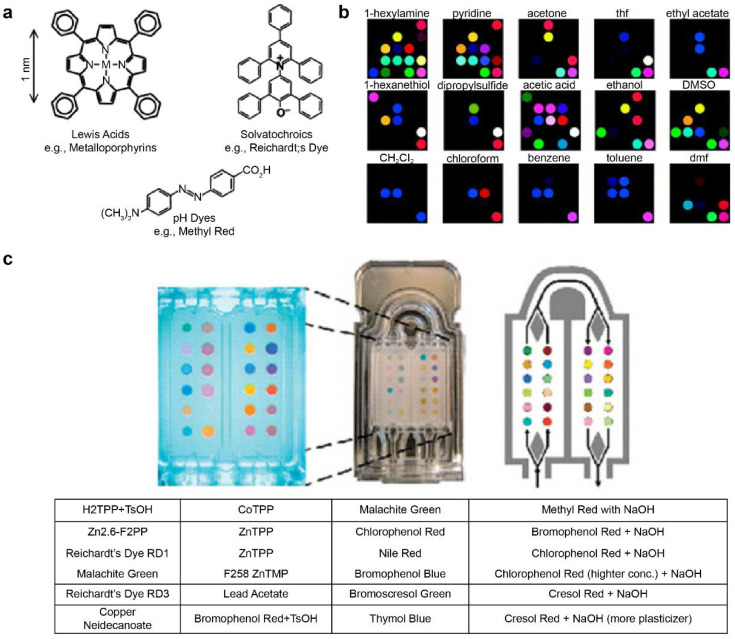
Metal-containing dye sensor array-type e-nose. (**a**) Suslick used strong chemical reactions such as “Lewis acid/base dyes (i.e., metal ion-containing dyes),” “Brønsted acidic or basic dyes (i.e., pH indicators),” and “dyes with large permanent dipoles (i.e., zwitterionic solvatochromic dyes).” Various types of sensor arrays were fabricated using the base dye (**b**) Electronic nose model that classifies various types of VOCs using the manufactured sensor array. (**c**) Exhalation analysis of lung cancer patients using the e-nose technology. The images (**a**,**b**) were adapted with permission from [130]. The image (**c**) was adapted with permission from [103].

The pattern information for specific reactions can be stored in the fingerprint form using metal-containing dye arrays that react at gas concentrations of hundreds of parts-per-billion. This technique is called smell seeding, and a number of studies focusing on the development of a personal chemical dosimeter for the detection, identification, and quantification of environmental and workplace VOCs have recently been published [123,124,125,126,127,128]. Furthermore, studies have been published on the development of medical diagnosis tools based on breath analysis. Rakow et al. fabricated a sensor array using the color transfer phenomenon of metal-containing dyes such as metalloporphyrin, which is sensitive to gas, and presented an artificial olfactory sensor model [129]. When there is a specific odor, the structure of metalloporphyrins and the color change (Figure 3a) [130]. Using 2D-displayed array metalloporphyrins, a pattern-recognition e-nose that detects a wide range of olfactants (including alcohols, amines, ethers, phosphines, phosphites, thioethers, and thiols) and weakly ligating solvent vapors (arenes, halocarbons, and ketones) was developed (Figure 3b) [130].

Mazzone et al. [103] analyzed exhaled breath samples from lung cancer patients using a colorimetric sensor array. The exhaled breath of 92 lung cancer patients and 229 control patients was obtained via the chromaticity sensor array. (Figure 3c) The technique is further expected to evaluate specific histologies of patients and to optimize them by incorporating clinical risk factors [103]. Other technologies for pathogenic fungal identification and rapid detection of bacteria have also been reported [131,132].

Kim et al. developed a superior chemical gas detection layer by simultaneously controlling nanostructures and catalytic functionalization [133]. The nanostructures derived from electrospinning act as highly dispersed ultrasmall catalysts. Electrospinning-derived 1D-dimensional MOs are nanomaterials with excellent advantages such as large surface-to-volume ratios, high porosity, and high gas permeability, which are beneficial for building chemical gas detection platforms. These materials are expanded to a variety of nanoarchitectures derived from metal organic frameworks, graphene oxide, and polymer templates, and they are used as a sensing sensor material [134,135,136].

Filamentous bacteriophage material is a functional bioreceptor with directed evolution (DE) properties (the Nobel Prize in Chemistry 2018) [137]. Kim et al. developed a multi-array sensor using a functional bacteriophage colorimetric sensor [138]. Phage-based colorimetric sensors classify various VOCs that can be used as an e-nose platform.

**Figure 4:**
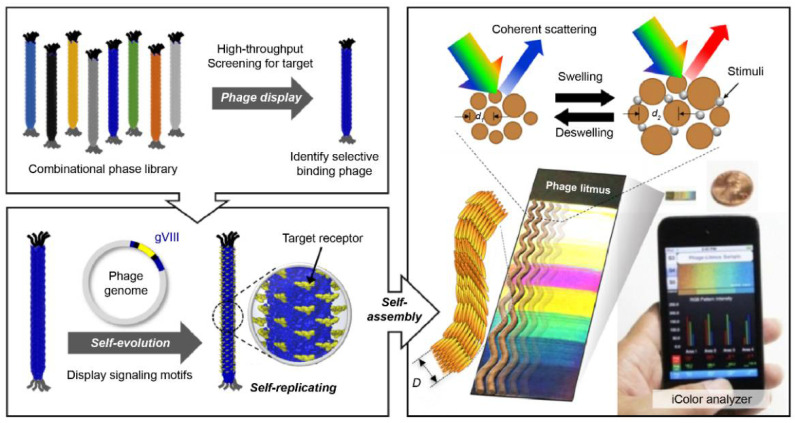
Colorimetric sensor system using an M13 bacteriophage as a functional biomaterial [109]. Peptide-based bioreceptor materials can secure various peptide sequences with desired functional groups using the phage display technology. Filamentous bacteriophage material with a scale less than 1 μm has approximately 2700 pairs of functional proteins on its surface. Hence, it can be used as a highly sensitive bioreceptor material. The synthesis process is based on internal DNA genetic information, and high-purity mass production is possible with simple genetic modifications. Phage units produced by bacteria have the same structure of a certain size and, thus have liquid crystal properties. A self-assembled structure can be produced using a bacteriophage as a unit, and it has liquid crystal properties; therefore, a color matrix can be produced by creating light scattering at regular distances formed by the structure. Based on the principle that phage self-assembled structures change the surface structure in response to external stimuli, a highly sensitive colorimetric sensor can be manufactured. This technique is referred to as the phage litmus.

Phage display technology, which is based on the principle of natural selection and proliferation by mutation, can discover functional bio-reporter peptide sequences with selectivity for specific molecules [139]. Through a simple genetic manipulation, it is possible to produce a functional phage material for a specific bio-reporter peptide discovered through phage display screening. The phage has ssDNA, which contains genetic information. It is synthesized through biological reactions and can be mass-produced as a bioreporter material with high-purity specific reactions. Oh et al. analyzed the bioreceptor function of the functional bacteriophage and presented the results of optical colorimetric sensor devices using the phage as a building unit (Figure 4) [110]. This single phage unit has a uniform fiber shape of less than 1 μm and has liquid crystal characteristics. Thus, a self-assembled nanostructure can be fabricated. This self-assembled phage structure forms a microstructure with quasi-ordered pitches and scattered reflected light [140]. The color of the phage structure changes according to the size and arrangement of the phage bundles. The external stimuli caused by chemicals change the arrangement of the bundles and change the color of the phage structure. These are used as a chemical gas sensor for VOC classification [109], food origin analysis [141], environment monitoring [142], and breath diagnosis [143]. Extensive research on the pattern-recognition integrated analysis technology utilizing various color sensor arrays is currently underway. Various studies have been published for the following five cell types: human hepatocellular adenocarcinoma (SK-Hep-1), cervical cancer (HeLa), human colon cancer (HCT116), human non-small lung cancer (NCI-H1299), and normal human embryonic kidney (HEK293); cells of these types were incubated in minimum essential media (MEM) containing 10% fetal serum culture [108]. Because each cell type produces a unique composition of VOCs, the three-band optoelectronic sensors produce a unique color. The results of the unique color change were obtained with 99.8% reliable data via 2D-linear discriminant analysis. The results of this study suggest the possibility of developing an effective diagnosis technology through further research.

## 4. Signal Processing Technology Based on Olfactory Cognitive Mechanisms

An artificial olfactory sensor is composed of a gas sensor array, which consists of multiple independent channels and an AI, which is an e-nose system that can detect odors and quantitatively measure their types and concentrations. Fundamentally, signal detection and data processing are required in the olfactory recognition mechanism. By leveraging various sensor arrays to construct multichannel reporters, signal data processing is required. An increasing number of signal receptors results in varying patterns of sensor responses, and a systematic data analysis algorithm using a multisensor array allows for trending classification. By utilizing the pattern analysis of the measurement data using the sensor array, various diseases can be detected, and the influence of external environmental factors (smoking, gender, age, etc.) can be minimized using the accumulated database. The improved analysis algorithm for the accumulated data results in a high commercialization potential of the artificial nose system. Table 3 summarizes various disease diagnosis research cases through data processing using existing sensor arrays.

### 4.1. Artificial Intelligence Data Processing-Based Multisensor Pattern Recognition

Multivariate analysis of gas mixture data is required for the analysis of sensor array data. Principal component analysis (PCA) is a method that is often used for visually distinguishing between the same sample groups in a plot. A PCA plot is a two-dimensional picture of data from which the data maximum variance can be obtained. The intelligent olfactory sensor can be implemented through advanced data processing techniques using AI and machine learning (ML) with pre-processed data. The supervised learning methods are mainly used to establish a functional relationship between the measurement space and classification elements [165]. In the past decades, many learning methods have been developed, for example, least squares regression (PLS), support vector machine (SVM), artificial neural network (ANN), decision tree (DT), and K-nearest neighbor (KNN). Among these, neural networks such as multilayer perceptrons (MLPs) have been widely used. Recently, the deep neural network (DNN) has been developed as part of a broader family of ML methods based on artificial neural networks. The ML is currently the most common application of AI and its principle is based on the automatic detection of patterns in data and these patterns can be used for future pattern recognition. It has become possible to derive new information by predicting or classifying collected data or by extracting information from appropriate data [166,167].

**Figure 5:**
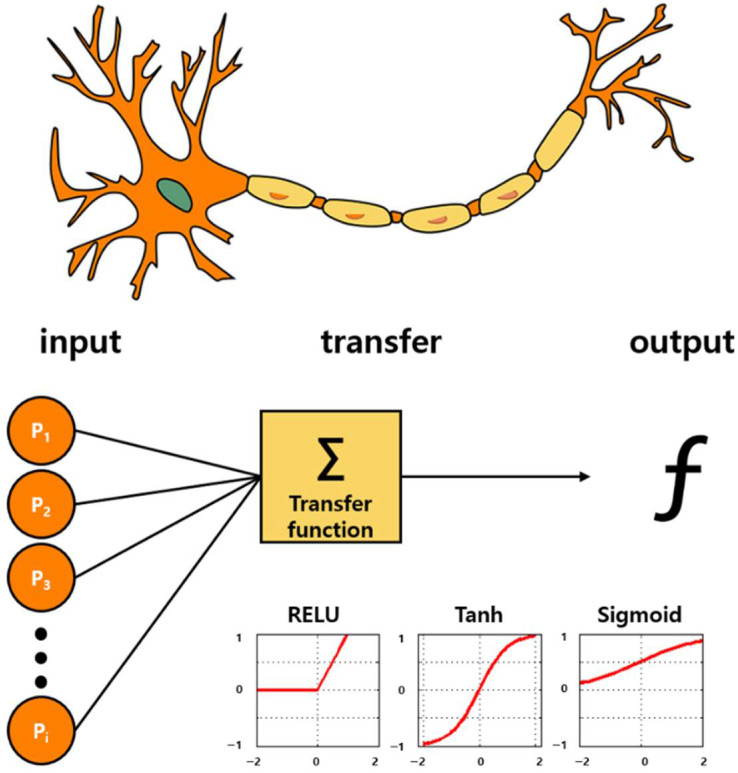
Bioinspired from neuronal pathway: Artificial intelligence algorithms. Deep learning technology based on the neuronal signal process. A structure that draws correct conclusions via iterative learning through activation functions called Sigmoid, Tanh, and ReLU. By applying ReLU to the inner hidden layer and by applying the sigmoid function in the last output layer, the accuracy is significantly increased.

Deep learning (DL) is also a branch of ML, and it is achieved through the ML based on neural networks. A neural network is inspired by biology, as shown in Figure 5, and it operates in the same way a biological brain solves problems with large units of axons connected to neurons. With the introduction of the concept of dropout, the over-fitting problem of neural networks was resolved, the accuracy was increased significantly, and the DL technology was newly highlighted [168]. This significantly reduced the computational time owing to the advancement of GPU hardware. In addition, the accuracy was considerably enhanced because accurate conclusions could be drawn through very fast iterative learning with big data. Recently, DL algorithms based on convolutional neural networks (CNNs) have shown excellent performance in various fields such as computer vision [169]. In particular, the performance of DL was further improved by the newly introduced ReLU activation function. The signal processing functions include sigmoid, tanh, and ReLU. The ReLU is a function that returns 0 when the value less than 0 is returned, and returns the same value when a value greater than 0 is found. The desired result can be outputted by applying ReLU in the internal hidden layer and sigmoid returning 1 if the value is greater than 0 in the output layer. This factor could be essential for the successful implementation of an intelligent olfactory sensor intended for the application in medical, environmental, and safety fields that require high performance based on big data [170,171,172]. Recently, several studies on the application of DL to intelligent olfactory sensors have been reported [173].

### 4.2. Multimodal (MM) Analysis

To analyze the concentration of a specific gas accurately, the influence of other gas components should be minimized. Several technologies are being developed for each sensor to minimize the effects of gases other than the gas component under analysis. However, few gases possess the same physical (mass, absorbance, etc.) and chemical (adsorption degree, reaction, etc.) properties. The gas components with the same chemical or physical properties cannot be separated using only a chemical or physical sensor. When a gas is analyzed based on several chemical or physical sensors, some measurement errors occur, and measurement sensitivity and reliability are not guaranteed.

By simultaneously analyzing measurement information of heterogeneous multimodal (MM) sensors, such as sensors that analyze chemical or physical properties, gas components that cannot be separated by the same type of the sensor method can now be separated. As a result, the gas analysis specificity can be improved. The ultralow-concentration (ppt or less) gases can be distinguished using MM information analysis based on measurement information of various kinds provided by pattern recognition-based sensors and AI technologies. The sensor-array that we introduced is also the multimodality concept. By arranging various sensors with different characteristics, each single sensor unit can complement each other against an external variable that cannot be distinguished. By extending the concept of multimodality, multi-array sensors of physical, chemical sensors, biosensors, etc. enables the identification of advanced information based on advanced discrimination.

The olfactory sensor technology is expected to develop into olfactory intelligence using pattern recognition, AI, and MM analysis. Consequently, the measurement sensitivity, accuracy, and reliability can be improved. Moreover, it is expected to develop into a future technology that provides new functions, such as early diagnosis/warning.

## 5. Conclusions

Clinical diagnosis and post-treatment monitoring technologies based on respiratory gas analysis have several advantages such as non-invasiveness, patient convenience, low cost, and real-time analysis. For successful implementation and use as a self-diagnostic device, this technology should be developed in the direction of miniaturization and simple sampling.

Organic compounds derived from the human body are indicators that assist with disease diagnosis. Body odor, sputum, urine, sweat, breath, etc. are the sources of odor required to analyze these organic compounds. Conductive polymer compounds with different physicochemical properties can be used to analyze the channel-specific pattern of electrical conductivity that changes depending on the type and concentration of gas molecules. Alternatively, olfactory receptors can be immobilized directly on each channel to provide specificity to gas molecules. In order for respiratory gas analysis to be established as a clinical examination tool, an in-depth and extensive clinical study of respiratory gas components and diseases should be conducted.

Olfactory sensors can be miniaturized and integrated using nanosensor technology, and they possess the advantage of low production costs. Currently, research is being carried out on the disease diagnosis analysis using various nanosensors. If a low-cost, easy-to-use, and portable artificial olfactory sensor is implemented, it can be extended and applied to all fields that require continuous monitoring. For the future development of diagnostic sensor technology in the form of an artificial olfactory perception model, multichannelling, miniaturization, weight reduction, low manufacturing cost, and sensor network construction are essential. Additionally, for the development of clinically reliable sensor array technology, a sensor element with high sensitivity as well as high selectivity performance should be developed, and a consistent breathing gas sampling method should be secured.

Advanced AI analysis methods such as DL are attracting significant attention because of their widespread use to improve the accuracy of signal analysis and result prediction of multichannel sensors in the future. Although the disease diagnosis using human breathing gas is still under development, a simple and convenient disease screening method using breathing gas has been designed through continuous gas analyzer development, sensor array technology development, and research on the relationship between human breathing gas and diseases. It is expected that this technology will be applied to disease screening in various forms, starting with the diseases.

Intelligent olfactory sensor technology holds potential for applications and services in various industries including medical, environmental, and safety fields that were previously impossible. In particular, the clinical equivalence and efficacy evaluation with existing medical devices for early disease monitoring using intelligent olfactory sensors in the medical field have been reported. Therefore, for the successful implementation of this technology, it is essential to develop a new technology that combines MM sensors that can provide high sensitivity, selectivity, and reliability with high-performance ML using big data.

## Figures and Tables

**Table 1:** Conventional measurements with the candidate group for biomarker volatile organic compounds (VOCs) in exhaled breath gas by a diagnosable disease. A simple and efficient e-nose platform that can distinguish VOCs in exhaled gas can be used as a selective self-diagnostic technology.

Disease	Conventional Measurement	Biomarker VOCs	Ref.
Diabetes	Glucose level Clinical biomarkers	Acetone	[24]
Bacterial infection	Computed tomography (CT) Gram stain Microorganism culture Morphological analysis High isoprene	Ammonia Hydrogen cyanide Nitric oxide Ethane Pentane	[18,25,26,27,28]
Asthma	Spirometry Peak expiratory flow Lung function testing Bronchoprovocation test	Acetone Nitric oxide Isoprene Ammonia	[29,30]
COPD	Spirometry X-Ray, CT Peak expiratory flow Lung function testing	Acetone Ethane	[31,32]
Cardiovascular disease (CVD)	HDL & LDL cholesterol High blood pressure Clinical biomarkers Obesity	Acetone Pentane Isoprene	[33,34]
Cancer	Clinical biomarkers Biopsy CT, X-ray, MRI	Acetone Formaldehyde Ethane Pentane Isoprene Ethanol	[35,36,37,38]

**Table 2:** Gas sensor technology applicable to future disease diagnosis.

Measurement Target	Sensor Type	Sensing Materials	Ref.
Lung cancer	Electrochemical sensor	Undoped SnO_2_, Co-SnO_2_, and Ni-SnO_2_ nanoparticles with cyclic voltammetry and electrochemical impedance spectroscopy/screen-printed electrode	[101]
	Colorimetric sensor	Colorimetric sensor array containing Lewis acid/base dyes (metal–organic complex dye)	[102,103]
Diabetes	Electrochemical sensor	Co_3_O_4_ thin film with a cubic spinel phase with AC impedance analyses/gold interdigitated electrode pattern	[104]
		Pristine SnO_2_ nanofiber (undoped) and Eu-doped SnO_2_ nanofibers (1, 2, and 3 mol% of Eu^3+^) with gold electrodes and Pt wires	[105]
Ethanol in a VOC mixture	Electrochemical sensor	CeO_2_–TiO_2_ core shell nanorods with Pt electrodes	[106]
		Pristine SnO_2_ and Yb-doped SnO_2_ hollow nanofiber (0.5, 1.0, and 1.5 wt% Yb) with an Au electrode and a Pt wire	[107]
Cancer cell culture	Colorimetric sensor	Functional M13 bacteriophage-based colorimetric sensor array	[108,109]

**Table 3:** Classification of disease groups through a data analysis algorithm using a sensor array.

Disease	Sensor	Data Process	Ref.
Lung cancer	Gold nanoparticle-based electrochemical sensor	PCA	[144]
Lung cancer cell culture	Cyranose^®^ 320	LDA, PNN, KNN	[145]
Lung cancer	Cyranose^®^ 320	SVM	[93,146]
Lung cancer and COPD	QCM sensor array	PLS-DA	[147]
Lung, breast, colorectal, and prostate cancers	Electrochemical sensor single array	PCA	[148]
Pulmonary disease	GC-MS/Chemo-nanoarray	DFA	[149]
Tuberculosis	BH114-Bloodhound	ANN	[150]
Urinary tract infections	BH114-Bloodhound	ANN, PCA	[151]
Brain cancer organoids	Polymer-carbon black based electro-chemical sensor array	Normalized pattern	[152]
Lung and gastric cancer, asthma and COPD	FET sensor	ANN, DFA	[153]
Renal dysfunction	Electrochemical sensor array	PCA	[154]
Gastric cancer	Aeonose	ANN	[155]
Gastric cancer	Metal–organic ligand-based nanosensor array	DFA	[156]
Pneumonia	Cyranose^®^ 320	PLS-DA	[157]
Ear, nose, and throat infection	Cyranose^®^ 320	PCA	[158]
Parkinson’s disease	Nanosensor array	KNN	[159]
Head and neck cancer	GC-MS	PCA, SVM	[160]
Human armpit body odor classification	Tagushi gas sensors	PCA	[161]
Colorectal cancer	GC-MS	DFA	[162]
Ovarian cancer	GC-MS	DFA	[163]
Seventeen types of diseases	Gold nanoparticle-based nanosensor array	ANN, hierarchal clustering analysis	[164]

PCA; Principal component analysis; LDA; Linear discriminant analysis; PNN; Probabilistic neural network; ANN; Artificial neural network; KNN; k-Neural network; SVM; Support vector machine; DFA; Deterministic finite automaton; QCM; Quartz microbalance; PLS-DA; Partial least squares discriminant analysis; Cyranose^®^ 320; a commercialized e-nose device, Cyrano Science, Pasadena, CA, USA; BH114-Bloodhound; a commercialized gas sensor array, Leeds, UK; Aenose; a commercialized e-nose device, The eNose Company, Zutphen, The Netherlands; FET; Field effect transistor.

## Data Availability

Not applicable. No new data were created or analyzed in this study.
